# Efficacy and Safety of Fospropofol Disodium for Injection in General Anesthesia Induction for Adult Patients: A Phase 3 Trial

**DOI:** 10.3389/fphar.2021.687894

**Published:** 2021-09-13

**Authors:** Chao-Meng Wu, Wen-Sheng Zhang, Jin Liu, Wei-Yi Zhang, Bo-Wen Ke

**Affiliations:** ^1^ Department of Anesthesiology, West China Hospital of Sichuan University and The Research Units of West China, Chinese Academy of Medical Science, Chengdu, China; ^2^ Laboratory of Anesthesia and Critical Care Medicine, Translational Neuroscience Center, West China Hospital of Sichuan University, Chengdu, China

**Keywords:** fospropofol, propofol, prodrug, general anesthesia, anesthesia induction, injection pain

## Abstract

**Background:** Fospropofol disodium for injection (Fospropofol_FD_) is a prodrug that is metabolized into propofol to produce a general anesthesia effect when administered intravenously.

**Objective:** This study aimed to assess the efficacy and safety of Fospropofol_FD_ in comparison with propofol medium/long-chain fat emulsion injections (propofol-MCT/LCT) for general anesthesia induction in adult patients undergoing elective surgeries.

**Setting:** Nine academic medical centers in China.

**Method:** This multicenter, randomized, double-blind, double-simulated, controlled, and non-inferiority trial evaluated 540 eligible adult patients randomly assigned (2:1) to the intervention (20 mg/kg Fospropofol_FD_) or control (2 mg/kg propofol-MCT/LCT) groups.

**Main Outcome Measure:** The primary efficacy endpoint was the success rate, defined as a Modified Observer’s Assessment of Alertness/Sedation Scale score of 1 within 5 min after study drug administration. The safety endpoints consisted of adverse events (AEs) related to consciousness, cognitive function, hemodynamic status, liver and kidney function, and blood tests.

**Results:** A total of 347 (96.3%) and 175 (97.2%) patients in the intervention and control groups, respectively, completed the study. The success rate for the primary outcome was 97.7% for both study drugs. The most frequent AEs in the intervention group were abnormal feeling (62.0%), blood pressure reduction (13.5%), and injection site pain (13.3%). No AEs related to consciousness and mental and cognitive functions or serious adverse events were reported.

**Conclusion:** Fospropofol_FD_ (20 mg/kg) is not inferior to propofol-MCT/LCT (2 mg/kg) in general anesthesia induction for American Society of Anesthesiologists (ASA) physical status I-II adult patients undergoing elective surgeries. It is safe and effective for clinical use under anesthesiologist monitoring.

**Impact on Practice Statement:** Fospropofol_FD_ can produce a general anesthesia effect and reduce the incidence of pain at the site of injection.

## Introduction

Propofol is an intravenous general anesthetic or sedative agent widely used in clinical procedures. Because it is highly lipophilic, its main formulation is an oil-in-water emulsion (Pardo and Miller, 2018). The advantages of propofol include its fast onset, short half-life, high clearance rate, and convenient target-controlled infusion. However, propofol is also known to cause adverse reactions such as injection site pain, thrombophlebitis, lipid metabolism disorders, bacterial infections, and allergy risks, which are considered to be associated with the oil-in-water emulsion formulation (Dinis-Oliveira, 2018). In some studies, less injection site pain in adults and teenagers was reported with medium/long-chain (MCT/LCT) preparations in comparison with long-chain (LCT) preparations (Larsen et al., 2001; Rau et al., 2001), but in other studies, the incidence and intensity of injection pain caused by MCT/LCT propofol were similar to those caused by LCT propofol (Knibbe et al., 2000; Krobbuaban et al., 2008; Varghese et al., 2010). Although some studies demonstrated that decreasing the free propofol concentration could reduce injection pain (Soltész et al., 2007; Yamakage et al., 2005), the occurrence of lipid metabolism disorders could not be completely avoided (Soltész et al., 2007).

Fospropofol disodium for injection (hereafter referred to as Fospropofol_FD_) is a prodrug of propofol with good water solubility that is manufactured by Yichang Human well Pharmaceutical Co., Ltd., Hubei, P. R. China. Fospropofol_FD_ is a sterile, non-pyrogenic, white or light yellow, lyophilized powder, and its chemical composition is 2, 6-diisopropylphenol methylal phosphate disodium salt hydrate. Each vial contains 500 mg of fospropofol disodium, which should be reconstituted with normal saline to a clear and colorless solution before intravenous administration. It does not require lipid emulsion as a drug carrier. This intravenous general anesthetic is metabolized into the active metabolite propofol, which is responsible for the pharmacological effect (Mahajan et al., 2012).

Phase 1 and phase 2 trials have demonstrated that intravenous Fospropofol_FD_ can induce an anesthetic effect with no serious adverse reactions (Yao et al., 2012; Yi et al., 2012; Liu et al., 2016). Based on phase 2 clinical trial data, we aimed to further assess the efficacy and safety of Fospropofol_FD_ 20 mg/kg in comparison with propofol medium/long-chain fat emulsion injection (hereafter referred to as propofol-MCT/LCT) 2 mg/kg for general anesthesia induction in adult patients undergoing elective surgeries in a phase 3 clinical trial.

## Materials and Methods

This multicenter, randomized, double-blinded, double-simulated, controlled, and non-inferiority trial was conducted at nine academic medical centers in China. This trial was designed to compare the efficacy and safety of intravenous injection of Fospropofol_FD_ with propofol-MCT/LCT for general anesthesia induction in adult patients. The study protocol was approved by the Medical Ethics Committee of the West China Hospital of Sichuan University and each participating center (Affiliated Hospital of Zunyi Medical University, Beijing Obstetrics and Gynecology Hospital of Capital Medical University, General Hospital of Tianjin Medical University, Guizhou Provincial People’s Hospital, Shanghai Changhai Hospital, The Second Affiliated Hospital of Wenzhou Medical University, The Second Xiangya Hospital of Central South University, and Wuhan Puai Hospital). This study was prospectively registered at the Chinese Clinical Trials Registry (http://www.chictr.org.cn/, registration number: ChiCTR1800016410). All participants provided written informed consent before participating in this study.

### Participants

Patients aged 18–65 years with body mass index (BMI) of 18–30 kg/m^2^ and American Society of Anesthesiologists (ASA) physical status I or II scheduled for elective surgical procedures under general anesthesia were enrolled in this study. The gender ratio of the participants was 1:1. The inclusion criteria were as follows: non-cardiothoracic, non-neurosurgery, or non-hepatobiliary elective surgery, estimated blood loss < 1,000 ml, duration of general anesthesia ≥ 30 min, requirement of endotracheal intubation, and preoperative electrocardiography (ECG), routine laboratory tests, and imaging within the normal ranges or abnormal results without clinical significance. The exclusion criteria were as follows: allergy to eggs, soy, and relevant products; allergic disease or allergy to the investigational drugs or drugs with a similar structure, contraindications to the investigational drugs; family history of malignant hyperthermia; pregnancy or breastfeeding, or a pregnancy planned within 1 month of the operation; systolic blood pressure (SBP) < 90 mmHg or >140 mmHg; heart rate (HR) < 50 bpm or >120 bpm; significantly prolonged QTcF interval with correction by the Fridericia formula (≥470 ms in men and ≥480 ms in women) and/or previous exposure to some QT prolongation drugs within 2 weeks before the screening period; known or predicted difficult airways; failure in the cognitive function exam using Mini-Mental State Examination (MMSE); coagulation disorders; history of alcohol or drug abuse; participation in other clinical medication studies (including the investigational drugs) within 3 months of the operation; sponsors or investigators or their family members who were directly involved in this study; or the investigators’ decision with no reason selected. Patients who met any of the above exclusion criteria were excluded. At each participating medical center, an independent preoperative investigator who was not involved in the subsequent study screened patients to identify eligible participants. The screening process was carried out from April 2017 to January 2018.

### Study Design

Based on the results of a phase 1 clinical trial (Yao et al., 2012; Yi et al., 2012), a Fospropofol_FD_ dose of 20 mg/kg was selected for comparison with 2 mg/kg propofol in a phase 2 clinical trial of the efficacy and safety of general anesthesia induction in patients with ASA grades I-II who underwent elective surgeries. The results demonstrated that Fospropofol_FD_ 20 mg/kg was non-inferior to propofol 2 mg/kg within 5 min after administration when used for inducing general anesthesia and was safe for clinical use by anesthesiologists (Liu et al., 2016). According to the CFDA requirements, a phase 3 trial would operate by expanding the sample size based on the phase 2 results (China Food and Drug Administration, 2003; Tian and Shao, 2010). Therefore, 20 mg/kg was also selected as the study dose of Fospropofol_FD_ to compare with 2 mg/kg propofol-MCT/LCT in this phase 3 trial.

An independent biomedical statistical company (Shanghai BioGuider Medical Technology Co., Ltd.) performed the randomization with a 2:1 ratio, block size of 6, stratified by the study center. The randomization was based on a central randomization system for clinical trials (DAS for IWRS 5.0, Beijing BioVoice Technology Co., Ltd.), and concealment of the allocation was ensured using password protection. According to the randomization sequence, an independent research nurse at each participating center who was not involved in the subsequent study prepared the study medications for both groups. Fospropofol_FD_, reconstituted with normal saline into an aqueous solution containing 5% (w/v, 50 mg/ml) fospropofol disodium, and normal saline placebo were prepared in identical syringes. Propofol-MCT/LCT (20 ml: 200 mg, Fresenius Kabi Company, China), a white, oil-in-water emulsion containing 1% (w/v) propofol, and 20% long-chain lipid emulsion placebo were prepared in identical syringes. For the purpose of double-blinding, the Fospropofol_FD_ group received Fospropofol_FD_ injection (0.2 ml/kg) and 20% long-chain lipid emulsion placebo (0.4 ml/kg), and the propofol-MCT/LCT group received propofol-MCT/LCT (0.4 ml/kg) and normal saline placebo (0.2 ml/kg). All participants, anesthesiologists, study investigators, and other healthcare providers were blinded to the group assignment and study medications.

On the day of elective surgery, the participants did not receive any preoperative medication prior to general anesthesia induction. After the participants entered the operation room, they were continuously monitored with standard 12-lead ECG and the pulse oxygen saturation (SpO_2_), respiratory rate (RR), blood pressure (BP), HR, body temperature (T), and bispectral index (BIS) were measured using multi-parameter monitors during the entire operation and post-anesthesia care unit (PACU) periods. The participants were required to be fully relaxed, and their vital signs were measured three times after consecutive oxygen inhalation (oxygen flow, 10 L/min) with a mask for 5 min. The average values were recorded as the baseline values of the vital signs. Two 18-gauge venous indwelling needles were each placed into the left and right median cubital veins of the participants’ arms, and a crystalloid solution (500 ml) was infused before induction.

Patients received the study medications for general anesthesia induction. Fospropofol_FD_ injection or normal saline placebo was administered via one median cubital vein by an attending anesthesiologist, and propofol-MCT/LCT or 20% long-chain lipid emulsion placebo was concurrently given via the other median cubital vein within 60 s by another anesthesiologist. Continuous and dynamic anesthesia evaluation was performed after the study drug administration, and the efficacy and safety indicators were recorded. When the participant’s Modified Observer`s Assessment of Alertness/Sedation Scale (MOAA/S) score reached 1 (Cohen, 2008), sequential intravenous injection of midazolam 0.04 mg/kg, fentanyl 3 μg/kg, and rocuronium bromide 0.9 mg/kg was initiated to complete the general anesthesia induction. Endotracheal intubation was performed at 2 min after combined administration. If the participant’s MOAA/S score failed to reach 1 within 5 min after the initial doses of the study drugs, a supplemental dose (half of the initial dose) was injected in both arms within a 30 s interval. If the MOAA/S score reached 1 within 20 min after the supplemental dose injection, the combined administration was continued for the participant, otherwise general anesthesia induction was considered to have failed and the patient was withdrawn from this study, after which the participant received additional induction to complete the surgery. Auxiliary/mechanically controlled ventilation was performed if respiratory depression/suspension was observed during the study.

For both groups, general anesthesia was maintained with a standard continuous propofol infusion (4–8 mg/kg/h, Propofol Injection, Diprivan^TM^, 500 mg/50 ml) and intermittent administration of midazolam (0.5 mg/h), adjusted to BIS values within the range of 40–60. Intraoperative analgesia was provided by remifentanil infusion (Remifentanil Hydrochloride for Injection, 1 mg diluted in 20 ml normal saline), adjusted based on the changes in HR and BP. The last dose of midazolam was given at approximately 1 h before the end of surgery. Propofol and remifentanil infusion were stopped at the end of surgery. All participants were extubated after surgery and observed in the PACU or the general or regular ward for at least 30 min until their full recovery. The follow-up interview was completed on the day after administration.

### Endpoints

The primary efficacy endpoint was the success rate with which an MOAA/S score of 1 was achieved within 5 min after drug administration. The secondary endpoints were the success rate in achieving an MOAA/S score of 1 within 25 min after administration, the supplementation rate of the study drugs, the length of time required to achieve an MOAA/S score of 1 after administration, the length of time to the loss of the eyelash reflex after administration, the BIS value, the length of time to anesthesia recovery, and the unit-time doses of propofol and remifentanil during maintenance of anesthesia.

The primary safety endpoints were the findings of central nervous system (CNS) assessments, including those pertaining to cognitive function, consciousness, and mental status; vital sign measurements (BP, HR, RR, and SPO_2_); ECG data; and the data for liver and kidney function. The secondary endpoints included the results of hematological and serum chemical assessments [electrolytes, glucose, total cholesterol (TC), triacylglycerol (TG), temperature (T)], and adverse events/reactions (AEs/ARs). CNS status was assessed at the baseline, 60 min after recovery of anesthesia, and in the follow-up interview. The vital signs and ECG were assessed at the baseline, during the surgery, in the PACU or the general or regular ward, and the follow-up interview. The laboratory tests were performed at the baseline and in the follow-up interview, and the AEs/ARs were observed and evaluated during the study period for each participant.

All data were stored electronically on a web-based database, and the principal investigator at each study center was responsible for ensuring data integrity. A supervisor and two research investigators from the lead study center (West China Hospital of Sichuan University) conducted site visits at the participating centers for data verification and collaboration during the study period.

### Statistical Analysis

The phase 2 clinical trial of Fospropofol_FD_ demonstrated that the results for the primary efficacy endpoint, i.e., the success rate in achieving an MOAA/S score of 1 within 5 min after drug administration, were 97.5% in the Fospropofol_FD_ group and 100% in the propofol group (Liu et al., 2016). Based on the literature review and clinical practice, clinical experts and statisticians decided that the non-inferiority value for this trial was −5% using a one-sided test, α = 0.025, and β = 0.1 (efficiency = 90%). All participants were randomly assigned (2:1 ratio) to the investigation group (20 mg/kg Fospropofol_FD_) or control group (2 mg/kg propofol-MCT/LCT) such that the sample size of the investigation group was 248 and that of the control group was 124. Considering the CFDA requirements (China Food and Drug Administration, 2003; Tian and Shao, 2010) and possible drop-outs (20%) during the trial, 360 participants in the investigation group and 180 participants in the control group were enrolled, with a gender ratio of 1:1.

All participants were randomly assigned to the investigation or control groups using a computer-generated randomization list (SAS 9.3 software, SAS Institute Inc., United States). Each center competitively enrolled the patients. The DAS clinical trial system (DAS for IWRS 5.0) for central randomization was used to apply random numbers and for dispensation of the study drugs.

In this trial, the statistical analysis dataset included a full analysis set (FAS), per-protocol set (PPS), and safety set (SS). The FAS was a case set of all randomized participants who had received at least one study drug and underwent at least one efficacy evaluation after administration. The PPS was a dataset consisting of all participants who had completed the study with the protocol. The SS was a safety dataset consisting of all participants who had received at least one treatment and had the actual data for safety endpoints recorded after treatment.

The primary efficacy endpoint was compared between groups using the chi-squared test, and the differences in rate and 95% confidence interval (CI) were assessed using the Wald and Newcombe methods. FAS and PPS analyses were both performed. Considering the preset non-inferiority value of −5%, the non-inferiority of 20 mg/kg Fospropofol_FD_ to 2 mg/kg propofol-MCT/LCT was concluded if the non-inferiority test yielded a *p*-value above 0.05 and the difference in the success rates for the primary efficacy endpoint was more than −5%. The secondary efficacy endpoints were analyzed in the FAS. The endpoints related to the success rate in achieving an MOAA/S score of 1 within 25 min after administration and the supplementation rate of the study drugs were compared between groups by using the chi-squared test. The continuous data were presented as mean ± standard deviation (SD) or median (interquartile ranges, IQR) and analyzed using the *t*-test or rank sum test, depending on the normality of the data distribution. The rank sum test was used to compare intergroup differences in the length of time required to obtain an MOAA/S score of 1 after drug administration, the length of time to the loss of the eyelash reflex after drug administration, the length of time to anesthesia recovery, and the unit-time dose of propofol and remifentanil during maintenance of anesthesia. The BIS values were compared with the *t*-test.

The safety endpoint analysis was performed using the data from the SS. The types, cases, frequencies, and incidences of AEs were calculated. Chi-squared test was used for comparison of the differences between groups.

In this trial, statistical analyses were performed by statisticians at Shanghai BioGuider Medicinal Technology Co. Ltd., China using SAS 9.4 software (SAS Institute Inc., United States). All statistical tests except those with the primary efficacy endpoint were two-tailed, and *p* values less than or equal to 0.05 were considered statistically significant.

## Results

### Study Population

**FIGURE 1:**
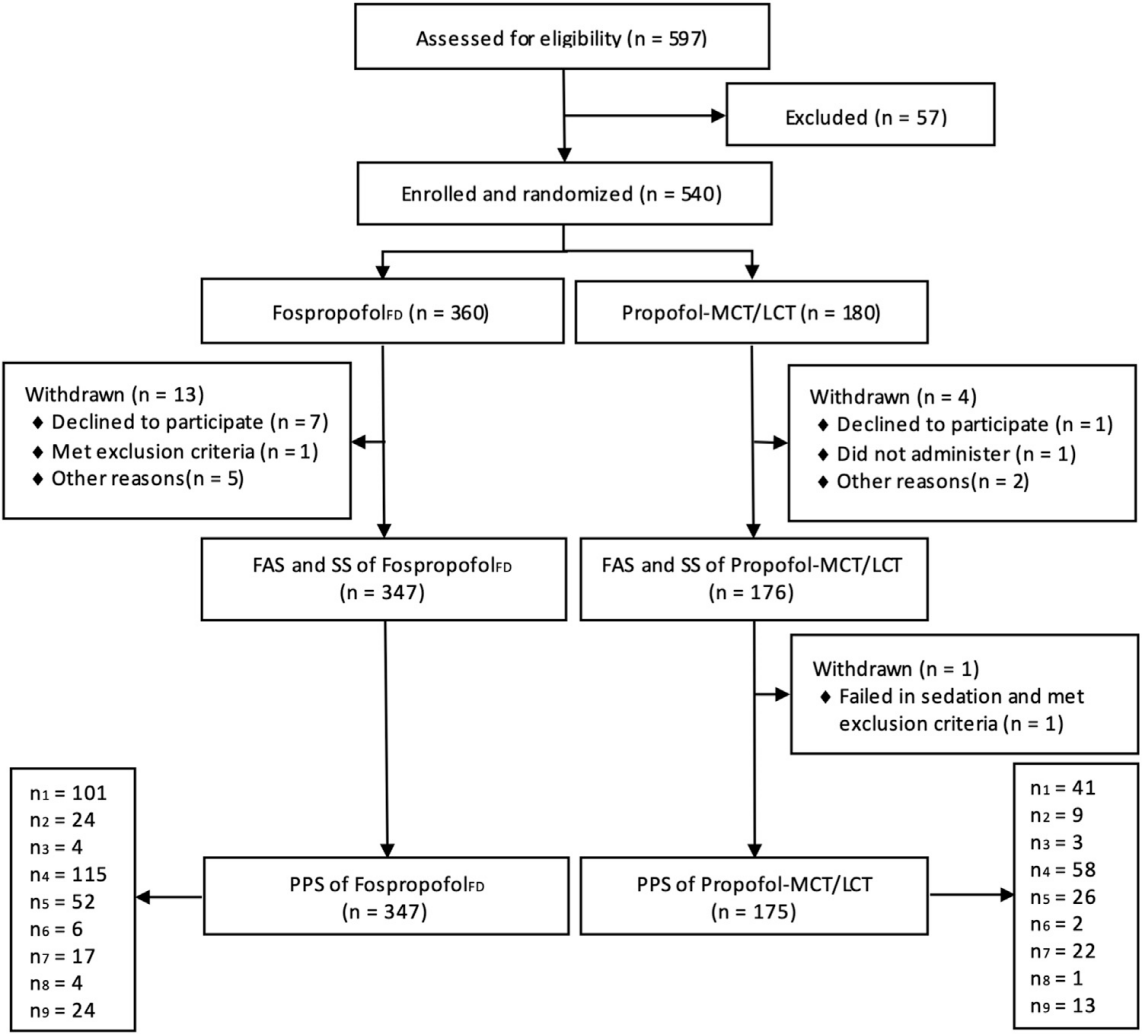
Diagram of participant flow (n_1_, n_2_, n_3_, n_4_, n_5_, n_6_, n_7_, n_8_, n_9_ indicates the number of participants in each research center).

Between april 18, 2017 and January 30, 2018, 597 patients were assessed for eligibility in nine academic centers. After excluding 57 patients who did not meet the eligibility criteria, a total of 540 participants were randomly assigned to the Fospropofol_FD_ (*n* = 360) or propofol-MCT/LCT (*n* = 180) groups. Of those, 522 participants (258 male) completed the study. One male patient in the control group withdrew from the study, because he failed to achieve an MOAA/S score of 1 within 25 min after study drug administration and concealed his history of drug abuse. The other 17 participants had withdrawn without any administration due to various reasons. Finally, the data for 523 participants were analyzed in the FAS and SS, and those for 522 participants were analyzed in the PPS. The study flowchart and the number of patients included in each study center are shown in Figure 1. The demographic characteristics and intraoperative information were comparable between the two groups (Table 1).

**TABLE 1:** Demographic characteristics and intraoperative information (FAS).

Characteristics	Fospropofol_FD_ (*n* = 347)	Propofol-MCT/LCT (n = 176)	*p* - value
Age (mean ± S.D., year)	36.9 ± 11.52	37.8 ± 12.63	0.39
Height (mean ± S.D., cm)	163.45 ± 7.85	163.45 ± 7.68	0.99
Weight (mean ± S.D., kg)	60.49 ± 9.79	60.00 ± 10.05	0.59
BMI (mean ± S.D., kg/m^2^)	22.56 ± 2.76	22.37 ± 2.78	0.46
Gender			
Male, n (%)	173 (49.9)	86 (48.9)	0.83
Female, n (%)	174 (50.1)	90 (51.1)	
Race			
Han, *n* (%)	309 (89.0)	157 (89.2)	0.96
Other, *n* (%)	38 (11.0)	19 (10.8)	
Education level			
Illiteracy	13 (3.7)	4 (2.3)	0.94
Primary school	51 (14.7)	31 (17.6)	
Middle school	145 (41.8)	70 (39.8)	
College and above	138 (39.8)	71 (40.3)	
History of past illness			
Yes, *n* (%)	144 (41.5)	68 (38.6)	0.53
No, *n* (%)	203 (58.5)	108 (61.4)	
History of procedures and anesthesia			
Yes, *n* (%)	180 (51.9)	88 (50.0)	0.69
No, *n* (%)	167 (48.1)	88 (50.0)	
ASA status			
I, *n* (%)	116 (33.4)	65 (36.9)	0.43
II, *n* (%)	231 (66.6)	111 (63.1)	
RR (mean ± S.D., bpm)	17.9 ± 2.77	18.3 ± 2.53	0.16
SBP (mean ± S.D., mmHg)	115.5 ± 11.05	117.7 ± 12.68	0.05
MAP (mean ± S.D., mmHg)	83.7 ± 9.49	85.2 ± 10.54	0.10
DBP (mean ± S.D., mmHg)	72.3 ± 8.74	73.1 ± 9.39	0.39
HR (mean ± S.D., bpm)	73.0 ± 11.84	72.0 ± 11.95	0.36
SpO_2_ (mean ± S.D., %)	99.8 ± 0.58	99.8 ± 0.59	0.87
T (mean ± S.D., °C)	36.48 ± 0.54	36.45 ± 0.56	0.65
BIS (mean ± S.D.)	92.51 ± 4.89	92.16 ± 5.96	0.51
Surgical procedures			
Otolaryngology surgery	101 (29.1)	52 (29.5)	0.71
Gynecological surgery	65 (18.7)	24 (13.6)	
Thyroid surgery	58 (16.7)	34 (19.3)	
Orthopedics surgery	64 (18.4)	31 (17.6)	
Vascular surgery	21 (6.1)	12 (6.8)	
General surgery	16 (4.6)	11 (6.3)	
Urologic surgery	10 (2.9)	9 (5.1)	
Oral surgery	6 (1.7)	1 (0.6)	
Anorectal surgery	3 (0.9)	1 (0.6)	
Gastrointestinal surgery	2 (0.6)	0 (0)	
Plastic surgery	1 (0.3)	1 (0.6)	

*S.D., standard deviation; SBP, systolic blood pressure; MAP, mean arterial pressure; DBP, diastolic blood pressure.

### Efficacy

The primary efficacy endpoint was the success rate in achieving an MOAA/S score of 1 within 5 min after administration. In the FAS or PPS, there was no statistically significant difference in the primary efficacy endpoint between the two groups (*p* > 0.05), and after adjusting for research center-related factors, the difference in the success rate between the two groups was more than the preset non-inferior value (−5%). The results indicated that the success rate in achieving an MOAA/S score of 1 within 5 min after administering 20 mg/kg Fospropofol_FD_ was non-inferior to that after administering 2 mg/kg propofol-MCT/LCT (Table 2).

**TABLE 2:** The success rate for MOAA/S scores that reached 1 within 5 min after administration.

Set	Group	Success	Not success	*p* - value	Difference (%)^a^	95% CI (Wald)^a^
FAS	Fospropofol_FD_, *n* (%)	339 (97.7)	8 (2.3)	>0.99	−0.41	−2.42 ∼ 1.63
	Propofol-MCT/LCT, *n* (%)	172 (97.7)	4 (2.3)			
PPS	Fospropofol_FD_, *n* (%)	339 (97.7)	8 (2.3)	0.76	−0.90	−3.34 ∼ 1.45
	Propofol-MCT/LCT, *n* (%)	172 (98.3)	3 (1.7)			

a After adjusting the research centers factors.

FAS, full analysis set; PPS, per-protocol set.

In the FAS, the success rates in achieving an MOAA/S score of 1 within 25 min after administration were 100 and 99.4% (*p* = 0.337) in the Fospropofol_FD_ and propofol-MCT/LCT groups, respectively; the supplementation rates for the study drugs were 0.9 and 2.3% (*p* = 0.232) in the Fospropofol_FD_ and propofol-MCT/LCT groups, respectively. The length of time to achieve an MOAA/S score of 1 after administration was equal to the length of time to the loss of the eyelash reflex, and this time was longer in the Fospropofol_FD_ group (median, 2.8 min) than in the propofol-MCT/LCT group (median, 1.42 min) (Table 3). These findings suggest that 20 mg/kg Fospropofol_FD_ had a slower onset than 2 mg/kg propfol-MCT/LCT.

**TABLE 3:** Secondary end-points (FAS).

	Fospropofol_FD_ (*n* = 347)	Propofol-MCT/LCT (*n* = 176)	*p* - value
Success rates of MOAA/S score reached 1 within 25 min after administration, *n* (%)	347 (100.0)	175 (99.4)	0.34
Supplemental rates of the study drugs, *n* (%)	3 (0.9)	4 (2.3)	0.23
Length of time to the loss of the eyelash reflex after administration (median, min)	2.80 (2.38~3.33)	1.42 (1.25~1.60)	<0.001
Length of time to MOAA/S score reached 1 after administration (median, min)	2.80 (2.38~3.33)	1.42 (1.23~1.58)	<0.001
Lowest value of BIS after administration (mean ± S.D.)^a^	19.26 ± 6.69	32.28 ± 9.06	<0.001
Length of time to the lowest BIS value after administration (median, min)^a^	13.0 (10.0~16.0)	4.0 (3.0~5.0)	<0.001
Length of time to anesthesia recovery (median, min)^b^	12.0 (8.0~19.0)	11.0 (7.0~15.0)	0.03
Unit-time dose of propofol during maintenance of anesthesia (mean ± S.D., mg/kg/h)	3.78 ± 1.47	5.38 ± 1.37	<0.001
Unit-time dose of remifentanil during maintenance of anesthesia (mean ± S.D., µg/kg/min)	0.12 ± 0.05	0.13 ± 0.04	<0.001

a The lowest value of BIS after administration was the lowest value which occurred before maintenance of anesthesia.

b Length of time to anesthesia recovery = the time of awake during anesthesia recovery – the time of anesthesia drugs stopping injection or surgery end time.

**FIGURE 2:**
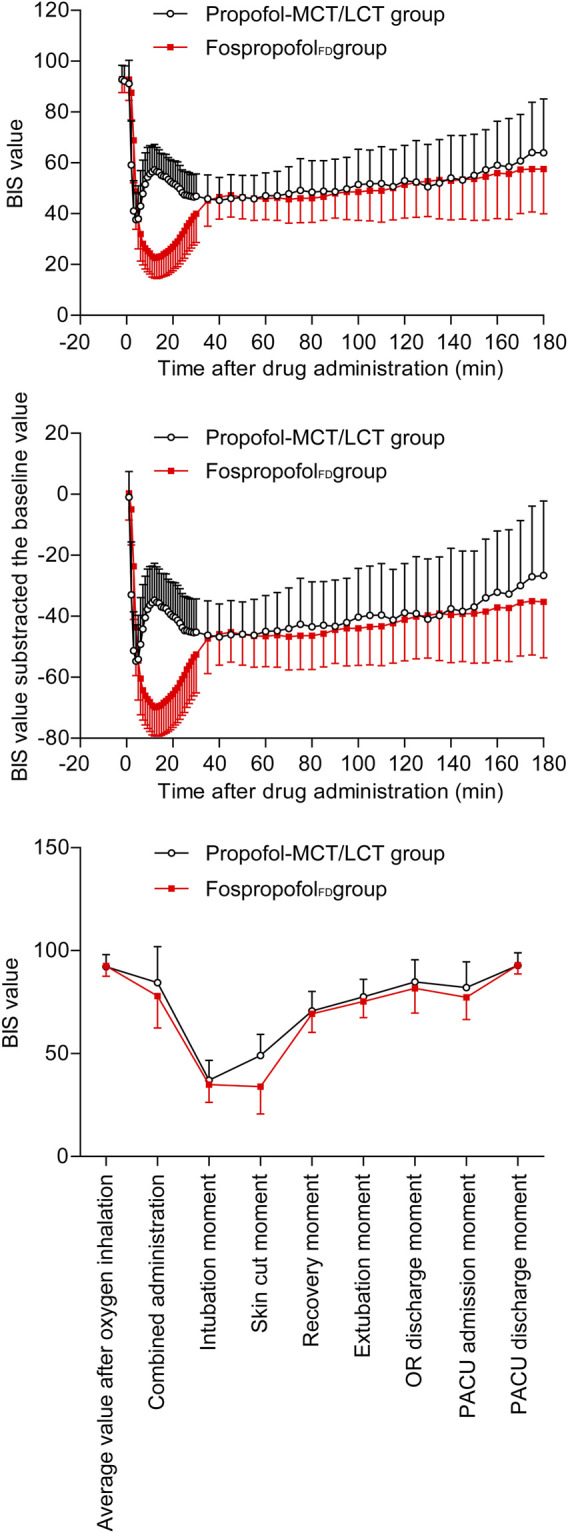
The BIS values.

The BIS values of the two groups decreased first and then increased after administration. Over the first 30 min after administration, the BIS values were significantly higher in the Fospropofol_FD_ group than in the propofol-MCT/LCT group at 1–4 min after administration (*p* < 0.05), similar at 5 min, but significantly lower at 6–30 min after administration (*p* < 0.05); the length of time to the lowest BIS value after administration was longer and the lowest value of BIS after administration was lower in the Fospropofol_FD_ group than in the propofol-MCT/LCT group (*p* < 0.05) (Table 3). However, the lowest BIS values in the two groups were both obtained after combined administration and could be affected by midazolam, fentanyl, and rocuronium bromide. The results indicated that 20 mg/kg Fospropofol_FD_ probably induced a deeper and longer sedative effect than 2 mg/kg propofol-MCT/LCT (Figure 2).

The duration of anesthesia in the Fospropofol_FD_ group (median, 110 min) was longer than that in the propofol-MCT/LCT group (median, 95 min) (*p* = 0.003). The length of time to anesthesia recovery in the Fospropofol_FD_ group was 1 min longer than that in the propofol-MCT/LCT group (*p* < 0.05) (Table 3), but BIS values at the time of anesthesia recovery were similar in both groups (Figure 2). During the maintenance of anesthesia, 21 (6.1%) participants in the Fospropofol_FD_ group and two (1.1%) participants in the propofol-MCT/LCT group did not use propofol, while three (0.9%) in the Fospropofol_FD_ group and three (1.7%) in the propofol-MCT/LCT group did not use remifentanil. The total doses of remifentanil (median, 750 μg) and midazolam (median, 0.5 mg) were not significantly different in the Fospropofol_FD_ and propofol-MCT/LCT groups, and the total dose of propofol (median, 455 mg) in the Fospropofol_FD_ group was less than that in the propofol-MCT/LCT group (median, 510 mg) (*p* < 0.05). However, the unit-time doses of propofol and remifentanil in the Fospropofol_FD_ group were both less than those in the propofol-MCT/LCT group (*p* < 0.05) (Table 3).

### Safety

In the SS, the incidences of ARs in the Fospropofol_FD_ group and the propofol-MCT/LCT group were 89.0 and 60.8% (*p* < 0.001), with the ARs mainly related to body sensation and the circulatory and respiratory systems. These were transient ARs that did not require any special treatment or could be relieved with routine treatments. Most ARs were mild to moderate in severity. No severe AR occurred in the CNS, circulatory system, and respiratory system. No serious adverse event (SAE) was observed. No participant was withdrawn from this trial because of any AR/AE.

There were 11 categories encompassing the 47 ARs in the Fospropofol_FD_ group, with the incidences of seven ARs being greater than 5%. The propofol-MCT/LCT group showed 30 ARs across 10 categories, with the incidences of five ARs being greater than 5% (Table 4).

**TABLE 4:** Adverse reactions (incidence> 5%) in the Fospropofol_FD_ and propofol-MCT/LCT groups (SS).

Preferred term, PT	Fospropofol_FD_ (*n* = 347)		Propofol-MCT/LCT (*n* = 176)	
	*n* (%)	frequency	*n* (%)	Frequency
Paresthesia	215 (62.0)	225	2 (1.1)	2
Hypotension	47 (13.5)	58	20 (11.4)	27
Pain on injection side	46 (13.3)	46	55 (31.3)	57
Aesthesia diminished	43 (12.4)	43	3 (1.7)	3
Respiratory depression	28 (8.1)	28	16 (9.1)	16
Apnea	25 (7.2)	27	17 (9.7)	17
Hypertension	22 (6.3)	39	16 (9.1)	24

The most common ARs in the Fospropofol_FD_ group were paresthesias (62.0%) such as pruritus, sensation of heat, electrical sensation, prickling, and biting, which were experienced in the perineal region, scalp, gum, lower body, or torso and were mainly mild (90.7%) in severity. The most common AR in the propofol-MCT/LCT group was pain at the injection site (30.1%), which was mild in severity in all cases. These two types of body sensation-related ARs were self-limited and transient and could be relieved before the consciousness state reached an MOAA/S score of 1 without any intervention.

No delayed recovery, cognitive dysfunction, or mental abnormalities were observed, and no intraoperative awareness occurred during the surgeries. These results indicated that the CNS-depressive effects of Fospropofol_FD_ and propofol-MCT/LCT were reversible, and the two agents had no significant effects on the brain function of the participants.

The pharmacological characteristics of the effect of Fospropofol_FD_ on the circulatory system were similar to the characteristics of its sedative effects. After administration, the BP and HR of both groups showed a minimal transient increase followed by a gradual decrease, and the intraoperative value was lower than the baseline until the anesthesia recovery period, when the value returned to the baseline level. After extubation, the BP and HR values were slightly higher than the baseline value, possibly due to many factors such as pain. The change trends for BP and HR in these two groups were almost the same.

In the intensive observation period within the first 30 min after administration, the values of SBP, diastolic blood pressure (DBP), and mean blood pressure (MBP) dropped below the baseline level at 4 min after administration in the Fospropofol_FD_ group and at 2 min after administration in the propofol-MCT/LCT group. The decrease in BP in the Fospropofol_FD_ group started later than that in the propofol-MCT/LCT group (*p* < 0.05). The values of SBP, MBP, and DBP in the Fospropofol_FD_ group were lower than those in the propofol-MCT/LCT group at most observation time points, and the extent of reduction was greater within 13 min after the administration but became comparable subsequently (Supplementary Figure S1). However, the incidence of hypotension as an AR was similar in both groups (13.5 vs 11.4%, *p* > 0.05). Thus, conventional treatment measures could relieve the hypotension.

In the Fospropofol_FD_ group, the extent of increase in HR at 1, 2, 5, and 6 min after administration was less than that in the propofol-MCT/LCT group (*p* < 0.05), and the extent of decrease in HR at 14–30 min after administration was also less (*p* < 0.05). These results indicated that the HR-depressive effect of Fospropofol_FD_ was weaker than that of propofol-MCT/LCT (Supplementary Figure S2). The incidence of low HR as an AR in the Fospropofol_FD_ group was 2.3% and that in the propofol-MCT/LCT group was 3.4%. All instances of low HR occurred after combined administration; thus, it is possible that the HR was affected by other drugs such as fentanyl. All instances of low HR recovered after observation or administration of atropine.

The incidences of ECG-related ARs were 13.3% in the Fospropofol_FD_ group and 12.5% in the propofol-MCT/LCT group, respectively (*p* > 0.05), with the ARs being ST-T abnormality (4.7 versus 4.6%), QT interval prolongation (3.5 versus 4.5%), sinus bradycardia (1.2 versus 2.3%), and sinus arrhythmia (0.3 and 0.6%). The ECG-related ARs did not cause any clinical syndrome and were relieved after observation without any treatment, except in two participants in the Fospropofol_FD_ group and one participant in the propofol-MCT/LCT group who used atropine intravenous bolus injection for sinus bradycardia.

The RR in both groups decreased after administration. There were no statistically significant differences in the incidence of respiratory depression (7.2 versus 9.7%, *p* > 0.05) and apnea (8.1 versus 9.1%, *p* > 0.05) between the two groups, and the respiratory depression mainly occurred during the induction periods. Auxiliary ventilation was easily performed by the anesthesiologist to maintain normal SpO_2_.

The body temperature of the participants decreased gradually 5 min after administration and increased gradually after the end of the surgery. The effects on temperature were comparable between the two groups (*p* > 0.05). No AR related to body temperature was observed.

The ARs in the laboratory tests were mainly related to abnormal liver function, such as abnormal changes in triglyceride metabolism (5.9 versus 5.2%) and transaminase (1.4 versus 0.6%) activity, all of which were mild in severity in both groups. The results of lab retests outside of the follow-up period showed improvement or normalization of the patients’ status. No ARs related to kidney function, glucose, cholesterol, electrolytes, inorganic phosphate, or hematological parameters were observed.

## Discussion

In this study, we observed the same results as those in the phase 2 clinical trial of Fospropofol_FD_, which showed that 20 mg/kg Fospropofol_FD_ had a slower onset and stronger and longer sedative effect than 2 mg/kg propofol-MCT/LCT (Liu et al., 2016). The main reason for this finding is that after intravenous injection into the human body, Fospropofol_FD_ is gradually metabolized into the pharmacologically active propofol, which produces an anesthetic sedative effect (Mahajan et al., 2012), whereas propofol-MCT/LCT produces an anesthetic effect directly. The clinical manifestation of Fospropofol_FD_ was consistent with its pharmacological properties (Mahajan et al., 2012; Yao et al., 2012; Liu et al., 2016). The secondary reason was that 20 mg/kg Fospropofol_FD_ probably became a higher dose when used in combination with other anesthetics for general anesthesia induction, although the lowest BIS values for the two groups were both obtained after combined administration. The median time to achieve an MOAA/S score of 1 in the Fospropofol_FD_ group was 2.80 min, which is acceptable for clinical general anesthesia induction. Nevertheless, the clinical anesthetic effect of intravenous induction and the quality of recovery in the procedure, which was no less than 30 min, were comparable to those obtained with propofol-MCT/LCT. Based on our results, the clinical implications of this study are as follows: 1) Fospropofol_FD_ is non-inferior to propofol-MCT/LCT in terms of the efficacy and safety, suggesting that it can be used in the routine practice of general anesthesia induction; 2) with a median onset time of 2.80 min, Fospropofol_FD_ may not be suitable for rapid sequence induction of general anesthesia, particularly in patients at a higher risk of reflux and aspiration; and 3) regarding the delayed offset of Fospropofol_FD_ when compared to propofol, Fospropofol_FD_ may be preferable for sedation in intensive care unit.

After administration of the study drug, the BP and HR in each group showed short elevations before decreasing. The main reason for this phenomenon was probably that the investigators needed to assess the consciousness of the participants through pain and speech stimulation while assessing the participants’ feelings. The nervousness of the participants, injection pain, and paresthesia might also be factors that influenced this finding.

In comparison with the propofol-MCT/LCT group, the extent of BP reduction was greater and the extents of HR elevation or reduction were smaller in the Fospropofol_FD_ group within 13 min after administration. This was probably due to the larger dose of Fospropofol_FD_ used (20 mg/kg), which induced a more significant vasodilation effect and a deeper anesthetic sedative effect. The participants’ stress response to stimulations such as tracheal intubation and position changes was lower. In comparison with the corresponding findings in the propofol-MCT/LCT group, the extent of HR reduction remained smaller in the Fospropofol_FD_ group after 14 min, but the extent of BP reduction in the Fospropofol_FD_ group was comparable between 14 and 26 min and became smaller after 27 min. The main reason for this result might be that propofol and remifentanil had to be used earlier and in larger doses to maintain the expected anesthesia depth in the propofol-MCT/LCT group.

According to the study protocol, lift mandibular or mask manual-assisted ventilation was required to resolve respiratory depression or apnea to ensure the participants’ safety. After tracheal intubation and mechanical ventilation, the anesthesiologists adjusted the ventilator parameters. Based on these intervention factors, the values of RR after administration were not analyzed, and only AEs were statistically analyzed. Similar to the results obtained in the phase 1 and phase 2 clinical trials of Fospropofol_FD_ (Yi et al., 2012; Liu et al., 2016), intravenous injection of 20 mg/kg Fospropofol_FD_ slowed RR and caused ARs such as respiratory depression or apnea, comparable to the findings obtained with 2 mg/kg propofol-MCT/LCT. However, anesthesiologists could easily resolve these ARs to maintain normal SpO_2_ and the recovery of postoperative spontaneous breathing was not affected.

Continuous dynamic monitoring of ECG was performed after administration. The cardiac physician diagnosed and interpreted ECG that was performed and recorded at multiple time points. QT interval prolongation was observed in both groups, although the prolongations were mild and scattered at different time points after administration in all cases. Anesthesia, surgical operation, electric knife usage, and electrolyte disorders after fasting before general anesthesia (including hypokalemia) can cause acquired QT interval prolongation (van Noord et al., 2010; Fazio et al., 2013), in addition to anesthetics that have a direct effect on ion channels and cardiomyocytes, e.g., propofol, succinylcholine, sevoflurane, desflurane, etc., which are commonly used for general anesthesia (Michaloudis et al., 1996; Heath and Terrar, 1997; Kleinsasser et al., 2000; Yildirim et al., 2004; Whyte et al., 2005). There was no continuous ECG acquisition and printing in this trial; thus, some cases of prolonged QT intervals might have been missed. However, no clinical symptoms of malignant arrhythmia occurred, and none of the cases of QT interval prolongation caused any clinical syndrome, and they all returned to normal without any special treatment.

Paresthesia was the most common AR in the Fospropofol_FD_ group, similar to the results of other domestic and international clinical trials, and was transient and self-relieving with no residual symptom (Bengalorkar et al., 2011; Cohen, 2008; Dinis-Oliveira, 2018; Liu et al., 2016; Yen et al., 2013). The total incidence of abnormal sensation (paresthesia, 62.0%; diminished esthesia, 12.4%) in this trial was less than that in phase 2 (95.0%) at the same dose of Fospropofol_FD_ (Liu et al., 2016). The Fospropofol_FD_ used in this trial was formulated with an improved production process. Hence, it probably contained fewer impurities and was more pure than that used in phase 2. Thus, we hypothesize that the increase in purity and the relatively larger dose of Fospropofol_FD_ caused the participants to enter a sedated state rapidly without being able to express or feel abnormalities. The abnormal sensation was probably related to the phosphorus produced by the intravenous injection of Fospropofol_FD,_ similar to other drugs that contain phosphate esters, such as dexamethasone (Perron et al., 2003; Cohen, 2008). No cases of abnormal serum inorganic phosphorus levels or renal dysfunction were observed postoperatively. Pretreatment with fentanyl before Fospropofol_FD_ injection could reduce the incidence of paresthesia (Cohen, 2008). The new phosphate-free water-soluble propofol prodrugs may also help prevent paresthesia (Lang et al., 2014). However, we still observed the same ARs (paresthesia, 1.1%; diminished esthesia, 1.7%) in the propofol-MCT/LCT group, suggesting that there might be other factors influencing the incidence of paresthesia and that further studies are needed.

Pain at the site of injection was reported by patients who received propofol-MCT/LCT (30.1%), Fospropofol_FD_ (9.2%), medium- and long-chain lipid emulsion (5.2%), or normal saline (3.4%). In these cases, the incidence of injection pain caused by emulsions was significantly higher than that caused by aqueous solutions (*p* < 0.05). Various methods are recommended to relieve injection pain, such as selecting large veins, using a slow bolus, adding lidocaine, and administering an injection analgesic before propofol-MCT/LCT or Fospropofol_FD_ (Jalota et al., 2011; Desousa, 2016).

In this trial, in order to observe and evaluate the effectiveness of 20 mg/kg Fospropofol_FD_ and 2 mg/kg propofol-MCT/LCT, we designed an administration protocol in which the study drugs were injected first, followed by injection of other sedatives, analgesics, and muscle relaxants after the patient achieved an MOAA/S score of 1. This method of general anesthesia induction was different from usual, and probably reduced the satisfaction of participants because of injection pain or paresthesia.

Cardiothoracic, neurosurgery, and hepatobiliary elective surgeries were not selected because of the need to minimize the impact of surgical procedures on safety assessment endpoints such as postoperative awareness and liver/kidney function. However, the study group will continue to explore the applicability of Fospropofol_FD_ in different procedures.

In conclusion, Fospropofol_FD_ (20 mg/kg) is not inferior to propofol-MCT/LCT (2 mg/kg) in general anesthesia induction for ASA I-II adult patients undergoing elective surgeries. It is safe and effective in clinical use under the monitoring of anesthesiologists.

## Data Availability

The raw data supporting the conclusions of this article will be made available by the authors, without undue reservation.

## Supplementary Material

Supplementary Figure S1 Comparison of BP between the two groups.

Supplementary Figure S2 Comparison of HR between the two groups.

## Glossary

AE: adverse events
AR: adverse reaction
ASA: American Society of Anesthesiologists
BIS: bispectral index
BMI: body mass index
ECG: electrocardiogram
BP: blood pressure
CFDA: China Food and Drug Administration
CI: confidence interval
CNS: central nervous system,
DBP: diastolic blood pressure
FAS: full analysis set
Fospropofol_FD_: Fospropofol disodium for injection
HR: heart rate
LCT: long chain
MBP: mean blood pressure
MCT/LCT: medium/long chain
MMSE: Mini-Mental State Examination
MOAA/S: Modified Observer’s Assessment of Alertness/Sedation Scale
PACU: post-anesthesia care unit
PPS: per-protocol set
propofol-MCT/LCT: propofol medium/long-chain fat emulsion injection
RR: respiratory rate
SAE: serious adverse event
SBP: systolic blood pressure
SpO2: pulse oxygen saturation
SS: safety set
T: body temperature
TC: total cholesterol
TG: triacylglycerol
