# Supplementary figures and images for: Efficacy and Safety of Fospropofol Disodium for Injection in General Anesthesia Induction for Adult Patients: A Phase 3 Trial
Source: Front Pharmacol. 2021 Sep 13;12:687894. doi: 10.3389/fphar.2021.687894 (PMC8473892; doi:10.3389/fphar.2021.687894)

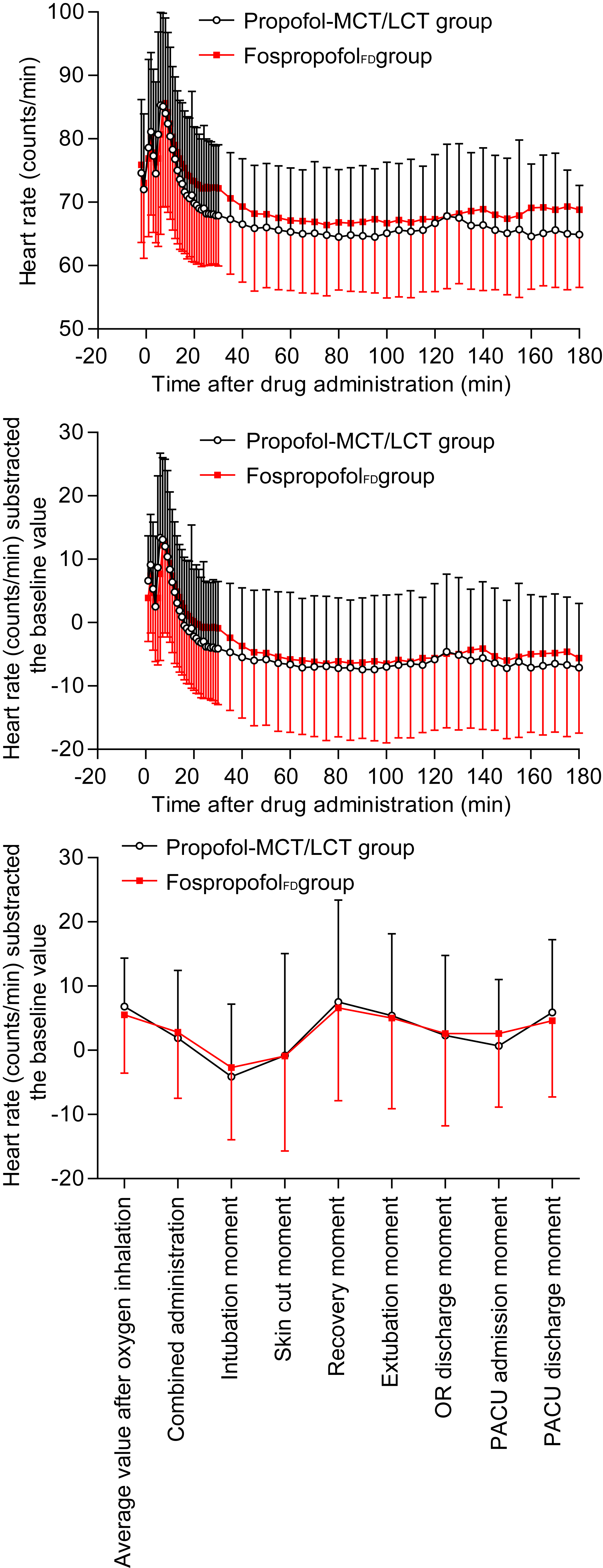

Supplement: Supplementary file 1 [file Image2.TIF]

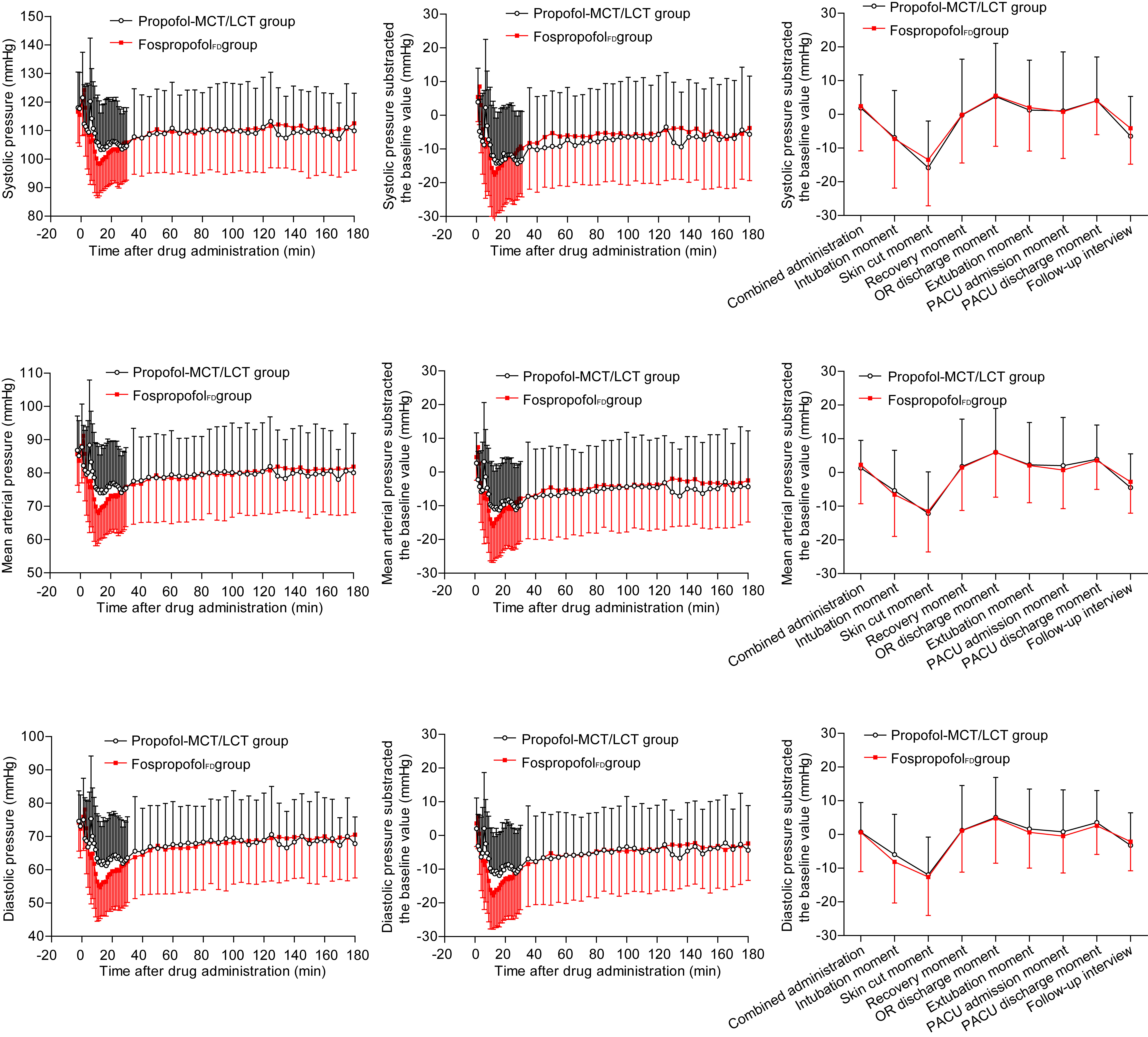

Supplement: Supplementary file 2 [file Image1.TIF]
